# Spatial Compression in Memory: How Repeated Walks on Familiar Routes Shorten Perceived Distance

**DOI:** 10.3390/bs15040404

**Published:** 2025-03-22

**Authors:** Kyung Wook Seo, Hyung-Sook Lee, Joo Young Kim

**Affiliations:** 1 Department of Architecture and Built Environment, Northumbria University, Newcastle upon Tyne NE1 8ST, UK; kyung.seo@northumbria.ac.uk; 2 Department of Landscape Architecture, Kyungpook National University, Daegu 41561, Republic of Korea; soolee@knu.ac.kr; 3 Siheung Research Institute, 11 Soraesan-gil, Siheung-si 14902, Republic of Korea

**Keywords:** cognitive threshold, information storage model, repeated journey, distance compression, spatial perception

## Abstract

Many experiments on distance perception have revealed that there is a difference between perceptual distance and objective distance. It has been accepted that a route with more memorable features will make its perceived distance longer. This study revisited this information storage model and examined how estimations change by repeated journeys in a university campus. While the outcome confirms the existing hypothesis, an unexpected pattern of distance compression by time was found. Spending more years on the campus, the estimation tended to decrease. The rate of decrease was bigger and more distinctively gradual for architecture and female students than non-architecture and male students. At the end, a cognitive threshold hypothesis was suggested as a possible model to explain the complexity of distance perception. Before reaching it, the distance grows along with the knowledge on a route but beyond the point of knowledge saturation, it begins to compress.

## 1. Introduction

It is widely known that our cognitive measure of the world is not aligned with the objective geometry of it. Numerous experiments have shown that human judgment of distance is influenced by the surrounding context and how it is remembered. Montello (1997) reviewed these experiments and organized the sources of distance knowledge into three classes: (1) number of environmental features, (2) travel time, and (3) travel efforts. He made it clear that the number of features has been proven to influence the judgment of distance, while travel time and efforts are not empirically proven as direct sources for it. Various types of environmental features have been tested in prior studies. In general, distance estimation is the function of the number of memorable features along the route. Any visual elements and meaningful information associated with it will affect the mental processing of retrospective distance assessment. Thus, a journey is perceived as longer with the increasing amount of features such as turns (Lee, 1970; Sadalla & Magel, 1980), intersections (Sadalla & Staplin, 1980), building facades (Nasar, 1983), visual relationships in the landscape (Coeterier, 1994), uphill or downhill roads (Okabe et al., 1986), or any imageable areas (Kaplan, 1973). This hypothesis will be called the information storage model in this paper, following the study by Sadalla and Staplin (1980). Then, how is it different from the first experience of the path when walks through it are repeated? We all remember the mysterious feeling of a new journey when it unfolded for the first time and how it gradually became established geographical knowledge in the following trips. Most of the studies outlined above, however, did not precisely take into account the level of subjects’ exposure to a route in their analysis. There exists research on the effect of multiple or return trips in the field of cognitive psychology, but they focus on the perception of time rather than distance (Roy et al., 2008; van de Ven et al., 2011; Hammond, 2012). One study could be identified to have tested the relation between the distance perception and the accumulative time spent on the route. In his experiment, Crompton (2006) observed that senior college students perceive the length of street segments longer than junior students; thus, distance grows along with the number of journeys. In his interpretation, students gradually grasp the details of streets near a campus as they spend more years at the university, and consequently the growing amount of features and memories make them perceive the same distance as longer. The experiment in this paper began by testing the generally accepted theory of the information storage model, but it was also hoped that Crompton’s unique approach could be tested in a different context.

## 2. Materials and Methods

**Figure 1:**
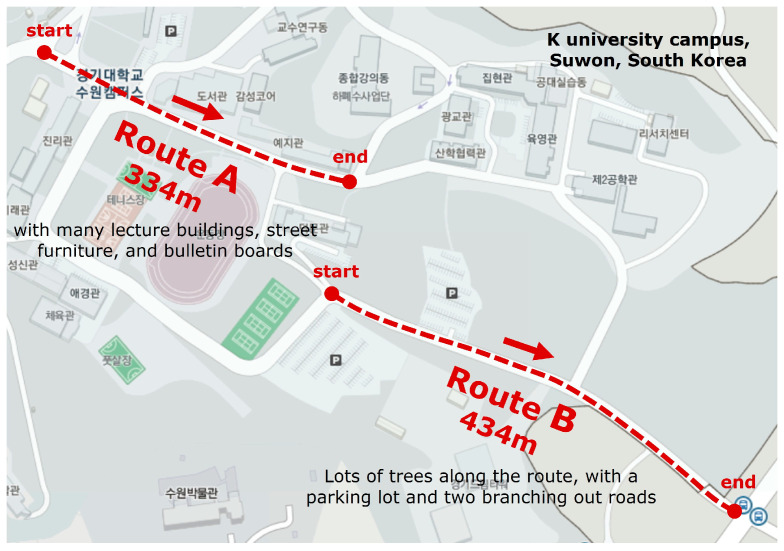
Two routes on K University campus.

The experiment was performed at the campus of K University in Suwon, South Korea. Questionnaires were distributed to undergraduate students in a lecture room on their retrospective mental estimations of selected routes. One survey was performed with architecture students (Sample 1), and to test the possible bias in the data caused by their major, a second survey was performed with non-architecture students (Sample 2). Two contrasting routes, A and B, were chosen to compare their perceived distance (Figure 1).

They both slope down toward the southeast direction with mild curves. Standing at one end, you cannot see the opposite end due to their curves. Route A starts from the main entrance of the campus at the top left corner in Figure 1 and ends at the student dining hall at its lowest. Along its 334 m journey, the slope has several gradient changes, and there are building facades on one side and outdoor bulletins, benches, and sports fields on the other. This route is regarded as a main street in the campus, which is populated by students and staff members constantly walking and interacting. Route B, by contrast, is off the central area of the campus, running 434 m down from the tennis court to the rear gate of the campus at the bottom right corner in Figure 1. Compared with the former, Route B is 100 m longer in its physical distance, which amounts to a substantial 30% additional length. It has a mild and continuous slope with roadside trees at regular intervals, and along the way there is no noticeable built structure except for a parking lot and two connection roads branching out from it. Thus, Route A is shorter but feature-loaded while Route B is longer but feature-sparse. Projecting their opposing conditions onto the information storage model, it can be assumed that Route A will be perceived as relatively longer than Route B. The two surveys with two sample groups were designed to check this hypothesis and its possible changes by repeated journeys.

## 3. Results

### 3.1. Sample 1 (Architecture Students)

Three groups of architecture students were recruited to investigate the information storage model. They were 57 first-year students, 49 second-year students, and 39 fourth-year students. At the time of the survey, the first-year students had spent 6 months on the campus, the second-year students had spent 18 months, and the fourth-year students had spent 42 months. Thus, the three groups provided distinct levels of experience in the campus; any senior group spends more time on campus, more than twice as long, than any immediate junior group.

**Figure 2:**
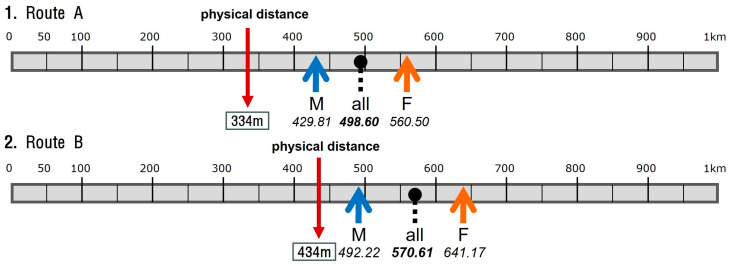
Distance estimations by 1st year architecture students.

The students were given a questionnaire with scale bars that showed 50 m intervals up to 1 km. They were then instructed to draw their mental estimation by arrows. Figure 2 illustrates the final summary of 1st year results marked on the same scale bars used for the questionnaire. The long red arrows indicate the physical distance of Route A (334 m) and Route B (434 m); the black dots indicate the overall averages (all); and the short arrows indicate the averages for males (M) and females (F). It is noticeable that the average estimations are all shifted to the right, with the overall average of 498.60 m for Route A and 570.61 m for Route B. As expected, the overestimation factor for Route A was bigger (1.54) than Route B (1.34). This means that first-year students’ mental image of Route A is 54% longer than it is in reality, compared to 34% for Route B. When the data are grouped by male and female students, an interesting split is observed. For both routes, females recorded far longer averages (560.5 m and 641.17 m) than males (429.81 m and 492.22 m). While the overestimation factor is still bigger for Route A, it was found that females tended to estimate each route as much longer than their male counterparts. This exactly follows what other preceding experiments reported; compared with their male counterparts, females have a stronger tendency towards overestimation (Nasar et al., 1985).

**Figure 3:**
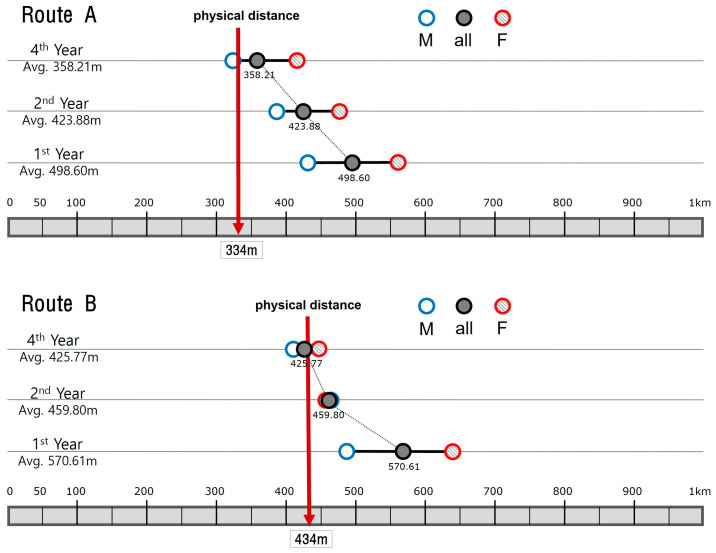
Distance estimations by 1st year architecture students.

Now, comparing the result of the first-year students with their senior counterparts, a striking pattern is found (Figure 3). Spending more time on the campus, the overestimation factor tends to decrease in a gradual manner. In the graph, as the year goes up, the overall average decreases towards the metric measure of the route. The estimation of Route A falls down from 498.60 m (1st years) to 423.88 m (2nd years) and then to 358.21 m (4th years), while Route B falls from 570.61 m (1st years) to 459.80 m (2nd years) and to 425.77 m (4th years). Hence, the ratio of the perceived distance against the physical distance decreased from 1.54 to 1.26 and to 1.06 for Route A and from 1.34 to 1.06 and to 0.98 for Route B. It is visually clear that in each year group, Route A is always estimated as proportionally longer than Route B, again confirming the information storage model. When the year groups are filtered by gender, another interesting pattern is revealed. In general, female students estimate the same physical distance as longer than their male counterparts within the same year group. This pattern is more pronounced for Route A where the sub-group averages for males and females flank the overall average on both sides in a consistent manner. The only exception is the 2nd year students on Route B, where the male average is slightly longer but almost identical to the female average. Statistically, however, this does not weaken the overall trend in perceived distance reduction over time.

**Figure 4:**
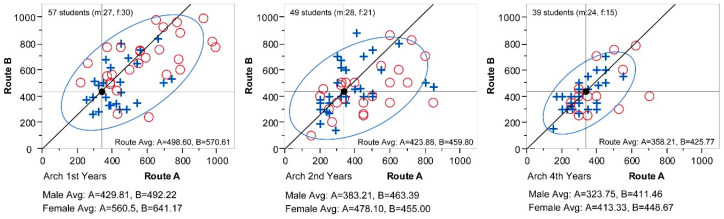
Individual estimations by architecture students (by year groups).

What has been observed so far is based on average values. There is always a danger of misinterpretation when a phenomenon is seen through the lens of mean values. To verify whether the previous pattern found by average values effectively represents the actual phenomenon, all individual answers are plotted on scattergrams in Figure 4.

Each of the three graphs show all the answers of the year groups (1st, 2nd, and 4th years, respectively), in which ‘+’ symbolizes male students, and ‘o’ symbolizes female students. Each point is coordinated by an x-axis for Route A and a y-axis for Route B. Thus, by looking at each coordinate point, it is possible to locate two perceptual differences by a student. The diagonal line in the graph delineates a gradient of 1.3, which is the physical distance difference ratio of Route B (434 m) to Route A (334 m), and thus passes though the coordinate (334, 434) marked as a black dot. With this line as a reference, answers positioned below it (or on its right side) can be interpreted as those who perceived Route A as relatively longer than Route B. The ellipse in the graph is a “bivariate normal ellipse” that shows 90% density of total values and as such effectively illustrates the territorial boundary of answers, i.e., where they are mainly clustered, by excluding 10% outliers. In all three graphs, it is observable that the dots are spread out farther to the right from the diagonal line, pulling the ellipses towards the same direction. Again, this conforms to the tendency of distance gain by loaded features in Route A.

When all three graphs are compared together, a meaningful pattern emerges. Moving from the 1st year students to the left towards the 4th year students, the bivariate normal ellipse shrinks and converges around the physical distance, x = 434 and y = 334. This follows the same pattern represented by average values in Figure 3. Spending more time on the campus, the distance becomes compressed proportionally. This result of estimation compression over time is quite surprising because it is completely the opposite to a similar experiment by Crompton (2006), in which he reasoned that the longer one experiences a route, the longer in distance it is perceived to be. His explanation was that its length mentally grows in proportion to the amount of detail one can remember. Then, why does the experiment in this paper show a contrasted outcome? There are subtle differences between this experiment and Crompton’s, but before opening a discussion on possible reasons for the incongruence, it is necessary to check the reliability of our experiment. Architecture students are trained to look at the environment with objective measures. More years of study could help develop a more accurate measurement system in their minds to see their surroundings. In this sense, the result from Sample 1 (architecture students), where the results of senior students approach the measurements of the actual physical distance, might be a reflection of their becoming trained architects, although Crompton’s experiment with students majoring in the same subject proved otherwise. To find out whether this is an exceptional case or something that can naturally happen, a group of non-architecture students were recruited to test possible pattern changes in distance perception.

### 3.2. Sample 2 (Non-Architecture Students)

The second survey was carried out by distributing the same questionnaire to students who enrolled in an elective lecture course on the same campus. The lecture was open to all university students, and a total of 284 students were asked the same questions. To draw an equivalent comparison with Sample 1, 3rd year students were excluded during the analysis; thus, the answers from 1st, 2nd, and 4th year students were used. Also excluded were seven architecture students for comparison, in order to ensure that the sample represented ‘non-architecture students’. Ultimately, a total of 229 students were included for the final analysis.

**Figure 5:**
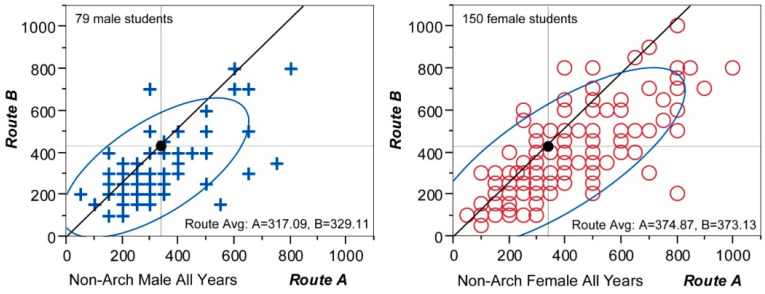
Individual estimations by non-architecture students (males and females).

The scattergrams in Figure 5 show all 79 male answers on the left and 150 female answers on the right. Although the number of female students is much bigger, they clearly display a difference, where female answers spread out farther towards the upper-right corner. The average distance estimations by males were 317.09 m for Route A and 329.11 for Route B, while those by females were 374.87 m and 373.13 m, respectively. Thus, the overestimation factors for Route A and B were 0.95 and 0.76 for males, compared with 1.10 and 0.84 for females. Again, all these results support existing findings of a female tendency to assume longer distance and a general tendency towards longer estimations for routes with more memorable attributes. The overestimation factors, however, were lower than those of the architecture students.

**Figure 6:**
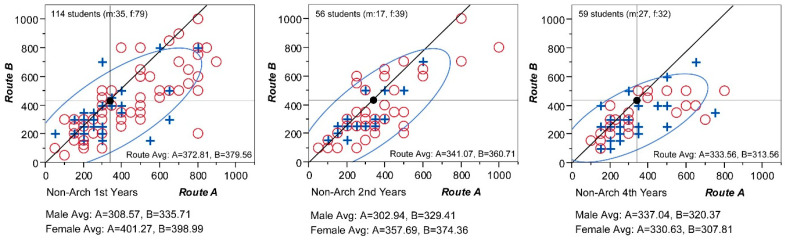
Individual estimations by non-architecture students (year groups).

**Figure 7:**
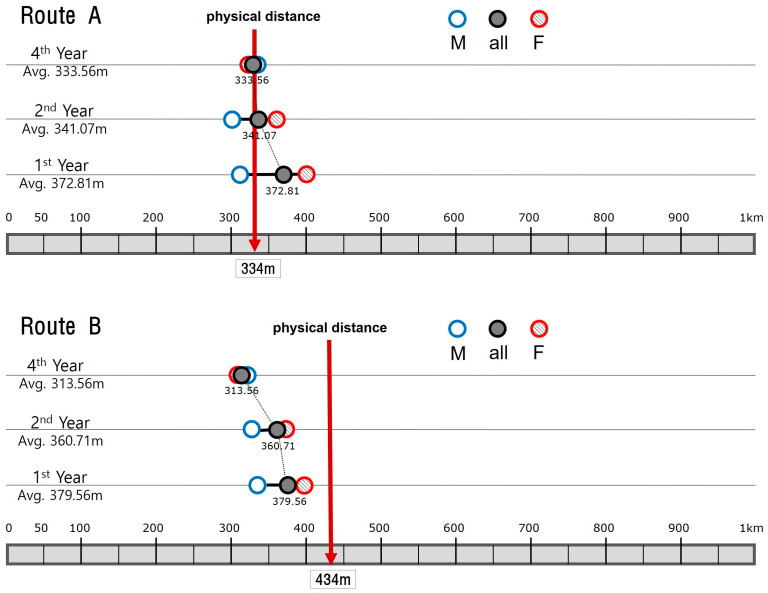
Estimations of non-architecture students compared by year groups and gender.

Then, the answers were regrouped according to the students’ years on campus (Figure 6). The 1st year group has 114 students, the 2nd year had 56 students, and the 4th year had 59 students. As before, ‘+’ indicates males, and ‘o’ indicates females in the graph. It is not as evident as in Experiment 1, but it is still noticeable that as the years increase, the distribution converges in such a way as to exclude outlying values, especially those in the upper-right section of the coordinate plain. In terms of average estimations, they too follow the pattern of gradual reduction summarized in Figure 7.

The perceived distance of Route A drops from 372.81 (1st years) to 341.07 (2nd years) and to 333.56 (4th years), while that of Route B drops from 379.56 to 360.71 and to 313.56. Undoubtedly, they show downward movement as the number of attendance years increases. When the year groups are divided by gender, it is found that female estimations indicate a clear decreasing pattern for both routes. In Figure 7, their estimates for Route A are 401.27 m (1st years), 357.69 m (2nd years), and 330.63 m (4th years), while those for Route B are 398.99 m (1st years), 374.36 m (2nd years), and 307.81 m (4th years). In comparison, male estimates show a complex pattern. While Route B conforms to the decreasing pattern, the reduction rate is relatively small, from 335.71 to 329.41 and to 320.37. The results for Route A, on the other hand, do not show a clear directional movement; its estimation decreases from 308.57 m (1st years) to 302.94 m (2nd years) but makes a sudden increase to 337.04 m (4th years). All the observations, taken together, still exhibit a general pattern of distance shortening as students become more familiar with the campus routes, although this pattern is less obvious than that of architecture students.

Comparing the two sample groups with different majors, the most prominent difference is that the estimations of the non-architecture group are considerably lower than those of the architecture group. By comparing their scattergrams in Figure 4 and Figure 6, it is evident that non-architects’ estimations are more densely plotted in the lower left corner, which is the zone where estimations for both Route A and B are shorter than the actual distances. Because of this, its bivariate normal ellipses further stretch towards the lower left corner, and this results in the estimation averages being much lower than those of the architecture group. In connection with the information storage model, this might mean that the distance measurement in the minds of architecture students is more finely calibrated than that of non-architecture students, possibly due to their trained ability to notice more details in surroundings. This probable discrepancy in spatial measurement according to academic education is worth looking into in future studies.

## 4. Discussion

Between the first and the second surveys, no prominent change took place in the campus environment to make the subjects perceive the routes differently. While both surveys confirm the existing theory of overestimation on information-loaded routes, as well as a tendency among females to estimate longer distances than males, they collectively point to a possible hypothesis: distance compression over time. Although the analysis contains some minor noise, it is more likely that a route would shrink along with the increased levels of experience and memory. Then, why did architecture students generate such a clean pattern than their non-architecture counterparts? It is vital for design career fields to develop skills to visualize objects and manipulate them through their minds and through graphic representations (Bilda & Gero, 2006; Berkowitz et al., 2020). Architectural institutions, in particular, provide courses to acquire these skills through 2D drawings and 3D modellings, enabling students to understand the built environment and surroundings in a systematic way (Arslan & Dazkir, 2017). Consequently, it is probable that students in architectural programs in the university use a more reliable approach to estimation, leading to more convincing results, compared with those in non-architectural programs. Unlike architecture students who continually learn how to design buildings and cities based on metric measures, the other group is not exposed to formal and consistent leaning of that kind. This could have resulted in their mental skill of distance estimation not changing as much as that of the architecture students in a consistent manner. Thus, the amount of time spent on campus was probably not as a critical factor influencing their perception.

In spite of this difference between the two groups, what has become clearer is that perceived distance does decrease over time in the context of our study. Now, an important question to answer is why this inverse relation of perceptual distance to time occurs in Suwon when other research reported a completely opposite finding in Manchester. In the Manchester experiment, Crompton gave instructions to architecture students to estimate distances in the same retrospective way as in this study. The difference was that students were asked to estimate each segment of the whole journey in an accumulative way. They were asked to make marks on a scale bar for each of the 22 destinations along the same straight route, 11 toward the city center from the campus and 11 away from it in the actual sequential order, and make adjustments afterwards. Unlike our experiment where a mental image of the route as a whole was recollected and estimated as a single entity, Crompton’s question was designed as a sum of segmentalized parts. In this way, it is highly probable that the estimate for each unit of destination was considered with more focus and care, encouraging students to remember more details during it. When a route is examined in this manner, students, in general, are likely to make longer estimations, and those with more experience have the potential to remember them as even longer. The result from his experiment, indicating that estimates grow over the years, could be translated along the lines of this questionnaire design. This plausibility can be supported by a segmentation model that posits that ‘a single estimate of an entire route’ would be shorter than ‘a sum of several segments’ of the same route, because longer segments are relatively underestimated while shorter segments are overestimated (Sadalla & Staplin, 1980; Montello, 1997).

In addition, route familiarity might have acted as another source of influence that caused the incongruity between the two results. Crompton’s experiment took the street that passes near the campus. Even though each segment of the route was short, about 160 m on average, the total distance stretched up to 2900 m (1.8 miles) from the starting point in each direction. By contrast, the routes we used were 334 m and 434 m, which are much shorter, and they were everyday passages inside the campus; thus, the students had a greater degree of familiarity compared to the off-campus Manchester routes. In fact, for attentive students, spending a semester on campus would be enough to remember all the buildings and other features along the routes. Therefore, the issue is not about how many buildings and details one can remember. Instead, how they are perceived in one’s mental simulation of walking through them becomes more important in estimation. When in a new urban setting, people often start to develop their spatial knowledge by adding landmarks, but as experience grows, the knowledge shifts from landmarks to ‘route’ and later to ‘survey knowledge’ (Platzer, 2005). Given that Route A and B in our experiment were relatively short and more frequently experienced as a whole, it is highly possible that students’ understanding of them followed this sequence rather quickly. What matters more, in this case, is how the physical experience of following the route is actually remembered. The physical experience of walking is closely linked to energy expenditure and environmental quality, and the former is related to walking time (Hsu & Tsai, 2014). Since the routes in our experiment were short, the duration of walking could have been a major factor in estimating their distance. It has been observed that the duration of time spent on familiar tasks is underestimated, while that of novel tasks is overestimated (Boltz et al., 1998; Hinds, 1999). Likewise, a meaningful correlation was found between the frequency of travel and perceived duration; i.e., a repeated trip makes travel time feel shorter (Roy & Christenfeld, 2007; Roy et al., 2008; Goldreich & Tong, 2013). If we accept the correlation between walking duration and distance perception, the distance compression over time in our experiment can be explained. Through countless repetition of the same journey for many semesters, the practice of following the route must have become a too-familiar activity, and consequently this could have led to a mental discount of the duration of walking the entire length of it. It might also have happened that students sometimes had to walk fast or run along the route to arrive at their class in time, and this in turn could have shortened the estimation of the lap time needed to complete the journey, especially for those who have spent a longer time on campus.

Now, embracing the theoretical assumptions above, a new hypothetical model can be suggested to explain the two conflicting results of distance shortening in Suwon and distance lengthening in Manchester. Imagine there exists a cognitive threshold of distance perception, a point of geographic knowledge saturation. Before reaching it, more trips can make the route perceived longer in proportion to the amount of meaningful knowledge acquired from it as in Crompton’s experiment. Moving beyond this point, additional walks will provide no further influential knowledge, serving only to make the journey feel easier without attentive effort, thus being less time-consuming and shorter. This model can also be applied to explain why distance estimates were shorter towards familiar destinations than towards unfamiliar ones in another experiment (Nasar et al., 1985). To test its plausibility, more fine-tuned experiments in various contexts would be required in future studies.

## 5. Conclusions

This research measured the degree of change in distance perception in relation to four variables, i.e., environmental conditions, gender, subjects’ majors, and the length of exposure to the environment. The results showed that the route with more imageable features was estimated as longer, and female students tended to estimate the route as longer than males did, confirming existing theories on distance perception. Splitting the answers by year groups, an unexpected pattern was found. Those who spent more semesters on campus felt the same distance to be shorter, and this pattern was more marked and consistent for architecture students and female students than non-architecture and male students. Surprisingly, this outcome of distance compression by time contrasted Crompton’s experiment, where senior students’ estimations were found to be longer than juniors’ estimations. Two strands of a theoretical assumption were made to explain this discrepancy. First, the segmentalization of the route in Crompton’s experiment might have caused the estimation growth by time. Second, the shorter routes in Suwon and their everyday use as main passages might have caused faster acquisition of the full details in the first-year students and thus shorter estimation of the lap time needed to complete the familiar journey for older students. Finally, it was suggested that there might be a cognitive threshold of informational saturation over which route perception can change in direction from lengthening to shortening. The contradictory findings of this research in comparison to Crompton’s experiment attests to why it is difficult and challenging to carry out experiments in a real-life environment. Unlike a reduced cue experiment in a controlled setting, our physical world carries with it a wide range of variables for each particular context. Whether distance perception grows or shrinks may not be easy to conclude in this respect, but it is hoped that subsequent studies in various environments will enable a more data-rich discussion.

## Data Availability

The raw data supporting the conclusions of this article will be made available by the authors on request.
